# Complex dynamics for a reduced model of human EEG: implications for the physiological basis of brain activity

**DOI:** 10.1186/1471-2202-12-S1-P198

**Published:** 2011-07-18

**Authors:** Dennis Buente, Federico Frascoli, David TJ Liley

**Affiliations:** 1 Mechanics and Ocean Engineering, Hamburg University of Technology, Hamburg, Germany; 2 Brain and Psychological Sciences Research Centre (BPsyC), Swinburne University of Technology, Australia

## 

**Figure 1:**
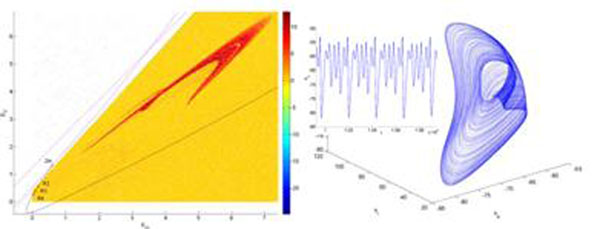
An example of complex behavior for Liley’s reduced model. On the left, resonant points (R2, R3, R4) emerge in a two parameters bifurcation diagram for changes in the thalamocortical input (p_ee_, p_ei_). These bifurcations give rise to quasiperiodic oscillations among different EEG bands. The superposed colored area is an evaluation of the dynamical activity for different values of (p_ee_, p_ei_), using maximal Lyapunov exponents. In a sea of (yellow) oscillatory patterns, a v-shaped chaotic area (in red) emerges. On the right, an example of attractor and its associated time series corresponding to such chaotic area is depicted.

Liley’s mesoscopic mean-field theory of mammalian cortex electro-rhythmogenesis describes the salient features of dynamical activity within a cortical macrocolumn based on the bulk interactions between inhibitory and excitatory neuronal populations [1]. Recently, a method for cataloguing the possible qualitative dynamical features of this model and other nonlinear systems of interest to neuroscience has been proposed [2], with the aim of establishing stronger connections between the dynamical patterns observed in neural field models and their neurobiological foundations. In this poster, a state space reduction of Liley’s model is briefly described with the behaviour of a number of different parametric instantiations illustrated to show that many of the important features of the original equations are preserved. The persistence of a richly complex dynamical landscape for this reduced model is apparent. For a range of physiologically meaningful values of parameters, it is found that local interactions between neuronal populations are sufficient to produce biologically compelling activity even in the absence of long-range cortico-cortical connections or spatial anisotropies. These patterns are either entirely novel or reproduce some of the known features of the full fourteen dimensional set of nonlinear equations in Liley’s model. Figure 1.

A number of highly nontrivial mechanisms for the birth of complexity are present in these reduced equations, among which we particularly note: i) chaos, generated by a Shilnikov saddle-node bifurcation, that acts as an organizing centre for the parameter space, ii) appearance of stable and unstable resonant bifurcation points responsible for quasiperiodic oscillations within EEG bands (e.g., alpha-gamma resonances), iii) existence of a number of oscillatory phenomena with biological relevance, such as mixed mode oscillations and multistability and iv) bursts and seizure-like dynamics for extended ranges of parameters. Based on this reduced model there are some speculative questions that we would like to propose in this poster. Firstly, is it possible to define a maximal number of degrees of freedom in neural field models that are sufficient to capture the relevant activity of mammalian cortex at rest? The high dimensionality and large parameter spaces of many of the existing models and theories of cortical dynamics discourages an in-depth, rigorous mathematical analysis, which are typically performed for models of spike generation. Secondly, can we be guided by reduced models in establishing a “hierarchy” of physiological causes for observed electrical brain patterns? And, finally, how do some of the findings presented here enrich our knowledge of the biological basis for rhythmic brain activity? For instance, our analysis points to resonances in human EEG being triggered by a physiological cause rather than being the product of complex synchronization mechanisms [3].
